# Multi-Substrate Specificity and the Evolutionary Basis for Interdependence in tRNA Editing and Methylation Enzymes

**DOI:** 10.3389/fgene.2019.00104

**Published:** 2019-02-14

**Authors:** Sameer Dixit, Jeremy C. Henderson, Juan D. Alfonzo

**Affiliations:** Department of Microbiology, The Ohio State Biochemistry Program, The Center for RNA Biology, The Ohio State University, Columbus, OH, United States

**Keywords:** translation, tRNA modification, mitochondria, inosine, deaminase, methylation

## Abstract

Among tRNA modification enzymes there is a correlation between specificity for multiple tRNA substrates and heteromultimerization. In general, enzymes that modify a conserved residue in different tRNA sequences adopt a heterodimeric structure. Presumably, such changes in the oligomeric state of enzymes, to gain multi-substrate recognition, are driven by the need to accommodate and catalyze a particular reaction in different substrates while maintaining high specificity. This review focuses on two classes of enzymes where the case for multimerization as a way to diversify molecular recognition can be made. We will highlight several new themes with tRNA methyltransferases and will also discuss recent findings with tRNA editing deaminases. These topics will be discussed in the context of several mechanisms by which heterodimerization may have been achieved during evolution and how these mechanisms might impact modifications in different systems.

## Introduction

Critical to substrate binding specificity is the fact that enzymes need to achieve “high” affinity for their targets while ignoring non-targets. What makes this an especially difficult problem in cells is that substrates and non-substrates often look very similar, which tests the limits of enzyme-substrate recognition. This tenet is especially true of RNA binding proteins where high-affinity binding usually involves indirect readout of the phosphate backbone of RNA that must be combined with base- and shape-specific contacts to enable substrate discrimination. Many enzymes follow these rules to achieve effective target specificity, yet some face an additional obstacle, in that they must also maintain the ability to turn over during a catalytic cycle in order to yield a productive reaction. This issue is exacerbated when a single enzyme must target different substrates within a pool of nearly identical ones, as is the case faced by most tRNA modification enzymes.

A growing trend in the modification field is that many of the enzymes which recognize multiple substrates are heteromultimeric (Guy and Phizicky, 2014), where partnering may contribute to higher binding affinity and enable discrimination between nearly identical substrates. In a previous model based on observations with tRNA deaminases, it was suggested that homodimerization was necessary for the bacterial adenosine to inosine (A-to-I) deaminase to recognize a single tRNA^Arg^ substrate (Ragone et al., 2011; Spears et al., 2011). On the contrary to accommodate multiple tRNA substrates of different sequences, key recognition motifs in the eukaryotic tRNA deaminases are positioned further from the active site (Ragone et al., 2011). Critically, such evolutionary adaptations would not have been possible without a move toward heterodimerization. In general, one could imagine that many heterodimeric (heteromultimeric) enzymes arose by gene duplication, which then allowed the duplicated genes to accumulate mutations, aiding in the process of neo-functionalization. Such is the case of the eukaryotic tRNA deaminase ADAT2/ADAT3, for which the two subunits are very similar but not identical, strongly arguing for a gene duplication event that led to functional differentiation of each subunit. A similar explanation may be true of other heteromultimeric modification enzymes, especially of methyltransferases. In the following pages, we will discuss in greater detail both the nature and evolution of heteromultimeric enzymes, with a focus on methyltransferases and deaminases. A previous review touched on the topic of neofunctionalization in the context of pseudouridine synthases (Fitzek et al., 2018). Here neofunctionalization will be only discussed in passing. We will highlight current themes and concepts that have arisen from recently published structures of a number of methyltransferases and also introduce the new concept of enzyme co-activation, whereby seemingly unrelated enzymes inactive on a specific substrate become active after association; a new twist to the idea of neofunctionalization.

## Interdependent Methyltransferases

### Methyltransferases That Require Heterodimerization

**FIGURE 1:**
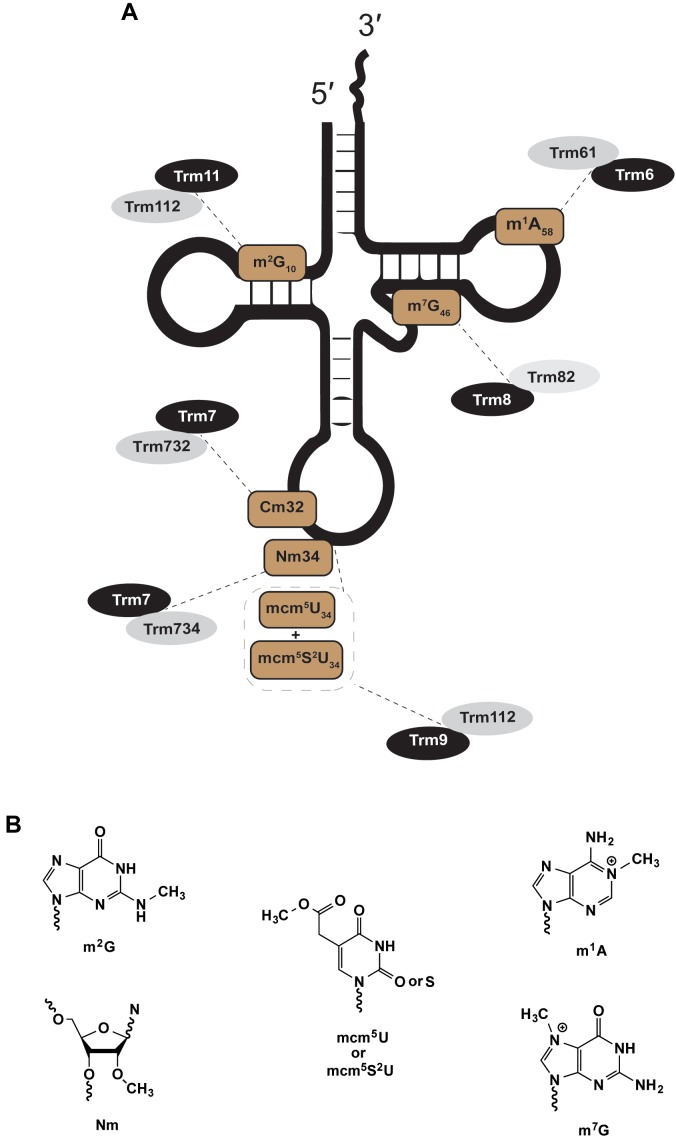
Eukaryotic two-subunit dependent methyltransferases. The figure shows examples of methylations catalyzed by heteromultimeric enzymes; a theme with eukaryotic modifications. **(A)** Shows the position and type of modification (brown) and the enzymes that catalyze such modifications (gray and black). **(B)** Chemical structures of modifications in **(A)**.

Certain eukaryotic tRNA methyltransferases strictly function as two-subunit enzymes: where association between a structural and a catalytic subunit is required for tRNA methylation (Figure 1). While this review will detail these associations, many recent reviews more broadly cover the enzymology and biological role of tRNA methyltransferase enzymes (Guy and Phizicky, 2014; Swinehart and Jackman, 2015; McKenney and Alfonzo, 2016; Goto-Ito et al., 2017; Hori, 2017; Traube and Carell, 2017). Beyond improvement to overall enzyme efficiency, heteromultimeric methyltransferases show altered activity toward particular tRNA species, particularly in choice of nucleotides and/or sites that are modified in a specific tRNA. In general, and where they have been identified, the equivalent bacterial and archaeal enzymes for a particular methylation do not require a heterologous partner for activity. Many have speculated that as the non-coding RNA pool expanded, pairing a non-catalytic with a catalytic subunit evolved to increase tRNA substrate specificity and prevent rampant non-specific methylation. Heteromultimerization may also integrate environmental and metabolic cues needed for optimal translation profiles that promote homeostasis; these possibilities will be explored further in the following sections.

#### Trm61/Trm6

The nuclear Trm61/Trm6 complex responsible for *N*-1 methyl adenosine at tRNA position 58 (Figure 1; m^1^A_58_) was first isolated from *S. cerevisiae* (Anderson et al., 1998, 2000). Trm61/Trm6 homologs exist in eukaryotic genomes of other yeast, protist, plant, and animal species (Bujnicki, 2001). The catalytic subunit Trm61 binds to co-substrate methyl donor *S*-adenosyl-L-methionine (SAM) through extensive hydrophobic and hydrogen bonding interactions (Wang M. et al., 2016). Trm61 association with Trm6 ensures high-affinity binding of Trm61/Trm6 to target tRNA substrates (Anderson et al., 2000; Ozanick et al., 2005, 2007; Finer-Moore et al., 2015; Wang M. et al., 2016). Disruption of Trm61 SAM binding eliminates m^1^A_58_ activity *in vitro* and *in vivo*, consistent with its assignment as the catalytic subunit (Anderson et al., 2000). Trm6 must associate with Trm61 for methyltransferase activity *in vitro* (Anderson et al., 2000), and *in vivo S. cerevisiae trm6* or *trm61* mutants lack m^1^A_58_-modified tRNA (Anderson et al., 1998). Human Trm61/Trm6 homologs can rescue the function of *S. cerevisiae trm61* or *trm6* mutants but only when expressed together, and maintain activity *in vitro* only when purified as a complex (Ozanick et al., 2005).

High-affinity binding of Trm61/Trm6 to tRNA substrates, such as tRNA_i_^Met^ or tRNA^Lys,3^_UUU,_ was originally thought to depend primarily on Trm6 as the RNA recognition subunit (Anderson et al., 2000; Ozanick et al., 2005). However, detailed mechanistic enzymology and crystal structures have refined this view, it is now clear that the Trm61/Trm6 holo-enzyme makes specific contacts with tRNA substrates (Ozanick et al., 2007; Finer-Moore et al., 2015; Wang M. et al., 2016). Together Trm61 and Trm6 create an L-shaped pocket to accommodate the tRNA substrate (Finer-Moore et al., 2015; Wang M. et al., 2016). The methylation site, nucleobase A_58_ is buried deep within the conventional L-shaped tRNA structure between D- and TΨC- stem-loop elements (Robertus et al., 1974). Crystallographic structure determination of the human Trm61/Trm6 complex bound to tRNA^Lys,3^_UUU_, revealed numerous protein–RNA interactions favoring separation of D- and TΨC- stem-loop elements, effectively allowing the enzyme to access the otherwise inaccessible *N*-1 of A_58_ (Finer-Moore et al., 2015).

Trm61/Trm6 has been suggested to arise through duplication and divergence from an ancestral TrmI-family enzyme, a family that catalyzes m^1^A_58_ formation in bacteria, and/or m^1^A_57_ in archaea (Grosjean et al., 1995; Bujnicki, 2001; Roovers et al., 2004; Ozanick et al., 2005, 2007; Guy and Phizicky, 2014; Finer-Moore et al., 2015; Wang M. et al., 2016). Purification of native TrmI from *Mycobacterium tuberculosis* and *Thermus thermophilus* yields stable TrmI homo-tetramers (Gupta et al., 2001; Droogmans et al., 2003; Barraud et al., 2008), a four subunit stoichiometry conserved in Trm61/Trm6, which forms a dimer of heterodimers (Ozanick et al., 2007; Finer-Moore et al., 2015). Many of the contact residues between TrmI subunits are maintained between Trm61/Trm6 subunits (Ozanick et al., 2007; Finer-Moore et al., 2015; Wang M. et al., 2016). The catalytic subunit Trm61 has high similarity to TrmI-family proteins, whereas Trm6 shows no apparent similarity to sequences deposited in current archaeal or bacterial databases. It is possible that Trm6 diverged from its ancestor, losing its ability to bind SAM, and maintaining only its RNA binding character. Interestingly, the tomato homolog of Trm6 (alias Gcd10) interacts with the dual methyltransferase/guanylyl transferase of the tobacco mosaic virus replicase complex (Osman and Buck, 1997; Taylor and Carr, 2000). This could indicate that other biologically relevant associations of Trm6 remain to be discovered. A recent report shows evidence that Trm61/Trm6 complexes catalyze low abundance m^1^A formation in regions of mRNA that loosely mimic tRNA TΨC-loops (Safra et al., 2017). A more detailed investigation of Trm61/Trm6 substrate specificity toward these mRNA structures is warranted.

#### Trm8/Trm82

The nuclear complex of Trm8/Trm82, responsible for *N*-7 methyl guanosine at position 46 (m^7^G_46_) in *S. cerevisiae*, shares many themes introduced in discussion of Trm61/Trm6 (Figure 1). Trm8 is a SAM-dependent methyltransferase that requires Trm82, a tryptophan-aspartic acid repeat (WD-repeat) homolog, for efficient activity (Alexandrov et al., 2002, 2005; Matsumoto et al., 2007; Muneyoshi et al., 2007; Leulliot et al., 2008). Deletion of either gene in yeast results in loss of m^7^G_46_ in tRNAs. Trm8/Trm82 can be purified as a stoichiometric complex, where co-expression is required for activity in cell-free wheat germ extracts (Matsumoto et al., 2008). Much like Trm61/Trm6, co-expression of the human homologs METTL1 (Trm8) and WDR4 (Trm82) restores m^7^G_46_ formation in Δ*trm8* or Δ*trm82* yeast strains, however individual expression of either human gene alone fails to complement these yeast mutants (Alexandrov et al., 2002). Disease mutants of either METTL1 or WDR4 that contribute to a rare form of primordial dwarfism, a prenatal growth deficiency that persists after birth, also result in human tRNAs that are deficient in m^7^G modification (Shaheen et al., 2015).

Trm8/Trm82 homologs are readily identifiable in yeast, protist, plant, and animal species. No apparent Trm8 or Trm82 homologs are currently found in archaeal genomes, which generally lack m^7^G_46_. Bacterial genomes do not contain an obvious Trm82 homolog, however, Trm8 shares sequence similarity with the bacterial TrmB-family (De Bie et al., 2003; Okamoto et al., 2004). Expression of TrmB homologs from *Aquifex aeolicus* or *Escherichia coli* in Δ*trm82* or Δ*trm8*Δ*trm82* yeast restores tRNA m^7^G_46_ formation without the need of an obvious cognate Trm82 (Alexandrov et al., 2005). This argues that Trm8 and TrmB may derive from a common ancestral protein, thematically consistent with the case of Trm61 and TrmI. However, unlike Trm61/Trm6, which may have arisen through duplication and drift, the partner subunit, Trm82, is a WD protein unrelated to any characterized methyltransferase.

Comparison of apo-Trm8/Trm82 to its tRNA bound form revealed that, unlike Trm61/Trm6, Trm82 makes no apparent RNA contacts in the context of a Trm8/Trm82 complex (Leulliot et al., 2008). This was corroborated by chemical crosslinking and small angle X-ray scattering experiments (Alexandrov et al., 2005; Leulliot et al., 2008). Although not formally tested, it is possible that Trm82 stabilizes conformations of Trm8 that promote substrate binding and/or productive methyl transfer. In turn, *in vivo* Trm8 levels are greatly reduced in Δ*trm82* yeast (Alexandrov et al., 2005), suggesting that Trm82 stabilizes cellular pools of Trm8 by a hitherto unknown mechanism.

#### Trm9/Trm112 and Trm11/Trm112

Trm112 can partner with either Trm9 or Trm11 to form separate SAM-dependent tRNA methyltransferase complexes, which act on separate pools of cytoplasmic tRNA substrates, at different nucleotide sites, and produce distinct chemical products (Figure 1). In *S. cerevisiae* Trm11/Trm112 forms *N*-2 methylguanosine at position 10 (m^2^G_10_) in a broad pool of tRNA species (Purushothaman et al., 2005; Okada et al., 2009). Trm9/Trm112 catalyze a more specific terminal methylation in the multi-step biosynthesis of 5-methoxycarbonylmethyl-2-thiouridine (mcm^5^s^2^U_34_) at the wobble position (position 34) in tRNA^Arg^_UCU_ and tRNA^Glu^_UUC_ in yeast (Kalhor and Clarke, 2003; Jablonowski et al., 2006; Begley et al., 2007), with additional tRNA isoacceptors in higher eukaryotes (Songe-Moller et al., 2010), including human tRNA^Lys^_UUU_ and tRNA^Sec^_UGA_. Trm9/Trm112 can also methylate non-thiouridine substrates to form mcm^5^U. The biological significance of yeast mcm^5^U-wobble site modification was assayed in a Δ*trm9* strain through comparative analyses of transcriptome, ribosomal footprinting and proteome data sets (Deng et al., 2015). Consistent with loss of their decoding function, hypomodified Trm9/Trm112 substrates tRNA^Arg^_UCU_ and tRNA^Glu^_UUC_, resulted in significant repression of protein expression for transcripts enriched in AGA or GAA codons (Deng et al., 2015). Analogous to two-subunit complexes discussed already, single yeast mutants of *trm112* or *trm9* each lack mcm^5^U_34_-tRNAs (Kalhor and Clarke, 2003; Mazauric et al., 2010; Chen et al., 2011), while *trm112* or *trm11* mutants each lack m^2^G_10_-tRNAs (Purushothaman et al., 2005; Okada et al., 2009).

Work in *E. coli* showed that co-expression of *S. cerevisiae* Trm9 and Trm112 is necessary to produce an active enzyme, as singularly purified catalytic subunit Trm9 is inactive (Mazauric et al., 2010; Chen et al., 2011). A crystal structure of the *Yarrowia lipolytica* Trm9/Trm112 complex, which shares 50–60% identity to the *S. cerevisiae* enzyme, has been reported (Letoquart et al., 2015). Co-crystal structures with substrate SAM or tRNA have not been obtained, although putative SAM-binding residues of the catalytic subunit (Trm9) have been mapped (Letoquart et al., 2015). The yeast Trm9/Trm112 association is stabilized in part by a β-zipper formed between parallel β-sheets of Trm9 and Trm112. Additional inter-subunit contacts bury a large hydrophobic region of Trm9, likely improving its solubility, and thus the *in vitro* methyltransferase activity of Trm9/Trm112 (Letoquart et al., 2015).

The C-terminus of the multi-domain protein methyltransferase ALKBH8 contains a subdomain with high sequence similarity to Trm9, sufficient in formation of mcm^5^U_34-_style modifications at the tRNA wobble position. ALKBH8 contains additional functional domains: an N-terminal RNA recognition motif, an AlkB-related domain, and a zinc finger region upstream to the C-terminal Trm9 orthology region (Fu et al., 2010; Songe-Moller et al., 2010; Leihne et al., 2011). Additional proteins with similarity to ALKBH8 exist in plant and protozoans, but many lack the Trm9-like methyltransferase domain (Zdzalik et al., 2014). ALKBH8 homologs with Trm9-like domains in *Mus musculus* and *Arabidopsis thaliana* maintain strict requirement for Trm112 association to form mcm^5^U-tRNA (Songe-Moller et al., 2010; Leihne et al., 2011), whereas the requirement for human ALKBH8 partnering with a Trm112 ortholog for wobble site methyltransfer has not been formally tested. Binding experiments have been performed, where substrate mimetic anticodon stem loop sequences bind more tightly to ALKBH8 in the presence of Trm112 (Pastore et al., 2012). Curiously, full length *in vitro* transcribed tRNA sequences showed no enhancement of ALKBH8 binding in the presence of Trm112 (Pastore et al., 2012). Specific examination of what impact the RNA recognition motif has on human ALKBH8 tRNA substrate specificity versus just the Trm9-like domain with Trm112 may prove insightful, especially as the substrate tRNA pool appears to have expanded in higher order eukaryotes. More detailed descriptions of molecular interactions between Trm9/Trm112 complexes and mcm^5^U-tRNA substrates remain outstanding.

The requirement of the *S. cerevisiae* Trm11 catalytic subunit to associate with Trm112 for m^2^G_10_ methyltransferase activity has been shown in recombinant, purified protein mixtures and wheat germ cell-free assays (Purushothaman et al., 2005; Okada et al., 2009) Currently, no structure of an intact Trm11/Trm112 complex has been reported. Eukaryotes and archaea have readily identifiable Trm11 homologs, which are otherwise absent from bacteria. An archaeal version of the catalytic subunit Trm11 from *Thermococcus kodakarensis* with bound SAM has been crystallized (Hirata et al., 2016). Detailed substrate interaction studies of yeast Trm11/Trm112 complexes showed that Trm112 improves Trm11 binding affinity for SAM and tRNA substrate (Bourgeois et al., 2017b). Whether Trm112 directly interacts with tRNA in the context of a Trm11/Trm112 complex, similar to Trm61/Trm6, or solely provides allosteric support for Trm11 tRNA binding, as proposed for Trm8/Trm82, remains an open question.

As previously noted Trm11 homologs are absent from bacteria, while Trm9 mcm^5^U-type modifications are exclusive to eukaryotes, yet Trm112 is broadly conserved across every domain (van Tran et al., 2018). Trm112 pairs with additional protein partners beyond Trm9 and Trm11 to form two-subunit methyltransferases whose substrates include ribosomal RNA or translation release factors, a topic well-reviewed elsewhere (Guy and Phizicky, 2014; Bourgeois et al., 2017a). In formation of active tRNA methyltransferase complexes it is likely that cellular Trm112 levels are limiting (Ghaemmaghami et al., 2003; Studte et al., 2008; Sardana and Johnson, 2012). Trm112 co-purifies stoichiometrically with Trm11 (Bourgeois et al., 2017b), and overexpression of Trm11 in yeast decreases the amount of Trm112 that co-immunoprecipitates with Trm9 (Studte et al., 2008). Biologically relevant conditions that depend on the competition between these catalytic subunits for Trm112 await discovery.

#### Trm7/Trm732 and Trm7/Trm734

The catalytic subunit Trm7, a 2′-*O* ribose methyltransferase, acts at nucleotides C_32_ or N_34_ dependent upon cytoplasmic association with partner subunits Trm732 or Trm734, respectively (Figure 1). Nm_32_ and Nm_34_ modifications are observed in all three domains. Eukaryotic tRNAs that contain Nm_32_ and Nm_34_ likely rely on Trm7/Trm732 or Trm7/Trm734 homologs, which are mostly but not entirely conserved throughout deposited sequences of eukaryotic genomes. Nm_32_ and Nm_34_ in archaea and bacteria are catalyzed by methyltransferases not obviously related to Trm7, Trm732 or Trm734. Nm_32_ modification is catalyzed by TrmJ-family members in bacteria and archaea (Purta et al., 2006; Somme et al., 2014), while TrmL forms Nm_34_ in bacterial tRNAs (Benitez-Paez et al., 2010) and box C/D small nucleolar ribonucleoprotein complexes form Nm_34_ in archaeal tRNAs (Clouet d’Orval et al., 2001; Nolivos et al., 2005; Joardar et al., 2011). One of these archaeal Nm_34_ modification complexes, was shown to use the excised intron from a processed tRNA^Trp^ transcript as the guide RNA to direct Nm_34_ of pre-tRNA^Trp^ substrates (Clouet d’Orval et al., 2001).

In yeast and other eukaryotes, certain tRNAs contain both Cm_32_ and Nm_34_ modifications in tandem. The most broadly conserved tandem methylated substrates are tRNA^Phe^ species. In yeast lack of Trm7-modified tRNA^Phe^ activates the general amino acid control starvation response (Han et al., 2018), whereas specific mutant lesions of the human ortholog FTSJ1 are linked to non-syndromic X-linked intellectual disability (Guy et al., 2015). Yeast form tandem Cm_32_ and Nm_34_ on additional substrates tRNA^Leu^_UAA_ and tRNA^Trp^_CCA_ (Guy and Phizicky, 2014). Evidence for the formation of separate Trm7/Trm732 or Trm7/Trm734 two-subunit complexes initially came from immunoblot pull down assays (Guy et al., 2012). Additional genetic and biochemical evidence support the hypothesis that Trm7/Trm732 or Trm7/Trm734 act as separate two-subunit complexes in 2′-*O* ribose methylation of C_32_ or N_34_ on yeast tRNA substrates, respectively (Guy et al., 2012). The predicted Trm7 ortholog in humans, FTSJ1, requires the cognate human homolog THADA, for Cm_32_ activity. However, *S. cerevisiae* Trm732 is able to functionally complement FTSJ1 in the absence of THADA (Guy and Phizicky, 2015). The Trm732 partner protein contains a conserved domain of unknown function as well as multiple armadillo-like helical domains, a structural fold generally important for protein and nucleic acid interactions. The other Trm7 partner, Trm734, is a WD-repeat protein similar to Trm82 the partner subunit of Trm8 m^7^G_46_ methyltransferase. More precise descriptions of interactions between subunits of Trm7/Trm732 and Trm7/Trm734 complexes, and of formed hetero-dimer complexes with substrate tRNAs, have yet to be articulated.

### Sequentially Ordered tRNA Methyltransferase Reactions

In most organisms, a tRNA sequence will contain well over a dozen post-transcriptional chemical modifications. An evergreen topic of discussion is whether modification enzymes act in a particular order, where modification at one site is informed by the modification status of other positions. In eukaryotes, compartmentalization of modification enzymes obviously results in the sequential order of some chemical transformations. For example, nascent tRNA transcripts are first modified in the nucleus; after export to the cytoplasm, tRNAs can be further modified. Because tRNAs can also be imported into organelles (chloroplast or mitochondria), these may receive other modifications in addition to those already obtained in the nuclear and cytoplasmic compartments. However, the sequential nature of certain tRNA methylation events cannot be explained by compartmentalization alone and may be an inherent property of enzymes that work in complexes or those who have achieved a heteromeric state. Alternatively, selection for increased specificity may be a leading factor in establishing sequentiallity, such may be the case of complex modification pathways such as those for wybutosine and threonylcarbamoyl synthesis.

With few exceptions, tRNA sequences encode for a purine at position 37 that is almost always post-transcriptionally modified. Guanosine at position 37 can be methylated, with more complex conversion to wybutosine (yW) in tRNA^Phe^ (Thiebe and Poralla, 1973; Noma et al., 2006). When adenosine is located at position 37 it may also be modified. In all three domains, threonylcarbamoyl can be found on N-6 of adenosine at position 37 (t^6^A_37_), or similarly isopentenyl (i^6^A_37_) modification may occur. In eukaryotic tRNA^Phe^ t^6^A_37_ and i^6^A_37_ are inhibitory of Trm7/Trm732 and Trm7/Trm734 Cm_32_ and Gm_34_ formation, and alternatively stimulate m^3^C_32_ formation by Trm140, or related enzymes in certain tRNA substrates (Guy et al., 2012; Arimbasseri et al., 2016; Han et al., 2017). In *E. coli* i^6^A_37_ similarly blocks the formation of Cm or Um at position 34, a reaction catalyzed by the TrmL family of enzymes (Han et al., 2017; Sokolowski et al., 2018). The extremophile *Thermus thermophilus* provides evidence for apparent temperature sensitive methylation circuits; at high temperatures the absence of m^7^G_46_ negatively impacts methylation at two other sites Gm_18_ and m^1^G_37_ (Tomikawa et al., 2010). A thematically similar report showed pseudouridylation at position 55 (Ψ_55_) impacts the formation of Gm_18_, m^1^A_58_, and m^5^s^2^U when cells are grown at lower temperatures (Ishida et al., 2011). Many more so-described sequential tRNA modification circuits exist beyond those covered here, and are reviewed elsewhere (Helm and Alfonzo, 2014; Maraia and Arimbasseri, 2017; Han and Phizicky, 2018; Sokolowski et al., 2018).

## tRNA Editing by Deamination

### Adenosine to Inosine (A-to-I) Editing

**FIGURE 2:**
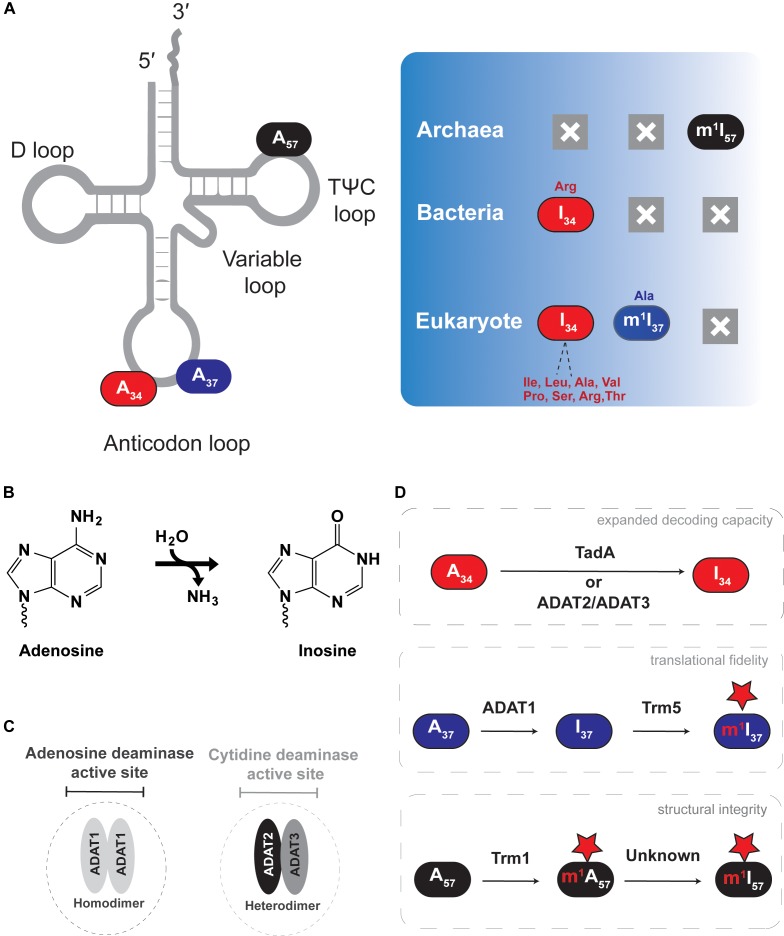
Adenosine-to-inosine (A-to-I) editing of tRNAs. The figure shows the different types of deamination reactions occurring in tRNAs from all domains of life. **(A)** Shows A-to-I edited nucleotide positions discussed in the text, with deamination end products and previous or subsequent methylation shown in the right panel. **(B)** Shows the general deamination reaction with ammonia as the leaving group and water as the key nucleophile. **(C)** Shows the characteristic active site of the different A-to-I deaminases. **(D)** Shows the effect each deamination has on tRNA function.

Chemical deamination of A-to-I in RNA sequences was observed 30 years prior to the discovery of any hydrolytic deaminase responsible for A-to-I activity that could act on polynucleotides (Bass and Weintraub, 1988). Since then, RNA adenosine deaminases have historically been divided into two broad classes based on their substrates: adenosine deaminases acting on mRNAs (ADARs) or adenosine deaminases acting on tRNAs (ADATs). Here we will focus exclusively on the tRNA deaminases (Figure 2). Inosine containing tRNAs are present in all domains of life; often tRNA A-to-I editing is essential. Unlike ADARs, which are generally promiscuous in A-to-I deamination of their targets (Bajad et al., 2017), ADATs show a more restricted A-to-I editing specificity, limited to three different tRNA sites: position 34 (wobble-position), 37 (3′ of the anticodon triplet) or 57 (in the TΨC-loop).

Inosine at the first position of the anticodon (I_34_) is essential in both eukaryotes and bacteria, but it does not occur in archaea (Figure 2). In eukaryotes, depending on the organism, roughly seven to eight cytoplasmic tRNAs have I_34_, while bacteria use I_34_ only in tRNA^Arg^ (Grosjean et al., 1996; Sprinzl et al., 1998). I_34_ increases the decoding capacity of tRNAs, allowing a single tRNA to decode three different codon (ending in U, C or A) and thus minimizing the number of necessary tRNA sequences that need to be genomically encoded. In addition, certain aminoacyl tRNA synthetases recognize and require the presence of I_34_ in tRNA substrates for productive aminoacylation (Droogmans and Grosjean, 1991; Senger et al., 1997; Gerber et al., 1998; Sprinzl et al., 1998; Losey et al., 2006). In bacteria, nearly all C-ending codons are read by tRNAs with an encoded G at position 34, except for tRNA^Arg^ which contains I_34_. Thus, despite a significantly more restricted pool of substrate tRNAs, I_34_ remains essential in bacteria.

A_34_-to-I_34_ deamination of eukaryotic tRNAs is catalyzed by the heterodimeric enzyme of (ADAT2/ADAT3 or Tad2/Tad3), which requires association between two paralogous sub-units for activity, while bacteria rely on the homodimeric enzyme ADATa (or TadA) (Auxilien et al., 1996; Wolf et al., 2002). *In vitro*, ADATa can efficiently recognize and deaminate a minimal substrate derived from the tRNA^Arg^ anticodon arm, while ADAT2/ADAT3 requires the entire tRNA for activity (Elias and Huang, 2005; Kuratani et al., 2005; Kim et al., 2006). Of special note, plant chloroplast also contains a single tRNA^Arg^ that undergoes A-to-I editing and relies on an ADATa-like enzyme, an observation consistent with the endosymbiotic theory of eukaryotic mitochondrial evolution (Delannoy et al., 2009; Karcher and Bock, 2009), but in general, mitochondria-encoded tRNAs do not contain inosine.

I_37_ and I_57_ are less widespread within organisms, where I_37_ is found in tRNA^Ala^ of certain eukaryotes (Gerber et al., 1998; Maas et al., 1999), and I_57_ has only been observed in tRNAs from archaea (Yamaizumi et al., 1982; Grosjean et al., 1995, 1996). Generally inosine at positions 37 and 57 can also be observed as methyl modified (m^1^I) as discussed later in Section “m^1^I Formation in Eukaryotes Versus Archaea.” I_37_ is formed by ADAT1, a homodimeric enzyme that shares key conserved residues with other deaminases: a conserved histidine and two cysteines that coordinate a catalytic Zn^2+^, as well as a conserved glutamate that participates in the final chemical step of inosine formation (Gerber et al., 1998; Gerber and Keller, 1999; Maas et al., 1999; Losey et al., 2006). I_37_ editing does not expand the decoding capacity of target tRNAs and its biological significance remains unclear. However, because of the importance of modifications at position 37 of the anticodon loop for reading-frame maintenance during translation, it is safe to assume a similar role for m^1^I_37_. The biological role of I_57_ in archaea is equally cryptic but its position in the backbone of the tRNA suggests a structural role.

All polynucleotide deaminases belong to the cytidine deaminase superfamily and require a Zn^2+^ for activity. However, phylogenetic analysis has revealed that ADAT1 closely aligns with mRNA specific ADARs, while ADAT2/ADAT3, responsible for inosine formation at position 34, strikingly resembles nucleic acid cytidine deaminases such as AID (activation-induced deaminases) or APOBEC (the apolipoprotein B mRNA editing enzyme) (Gerber and Keller, 2001). Recent investigation of the fungus *Fusarium graminearum* provides evidence that ADARs may have evolved from ADAT-like enzymes, as *F. graminearum* lack an obvious ADAR homolog, yet still contain A-to-I edited mRNAs (Wang C. et al., 2016; Bajad et al., 2017). These authors propose that ADATs may be responsible for A-to-I during the sexual life cycle of *F. graminearum*, which further suggests the double-stranded RNA binding domain found in ADARs was gained during the evolution from an ADAT-like ancestor, which lacks this domain (Gerber et al., 1998; Gerber and Keller, 1999; Maas et al., 1999; Losey et al., 2006). If true, it is equally possible that certain annotated ADATs may deaminate RNA substrates other than tRNAs, perhaps in complex with additional factors.

### Cytidine to Uridine (C-to-U) tRNA Editing in Archaea and Eukarya

**FIGURE 3:**
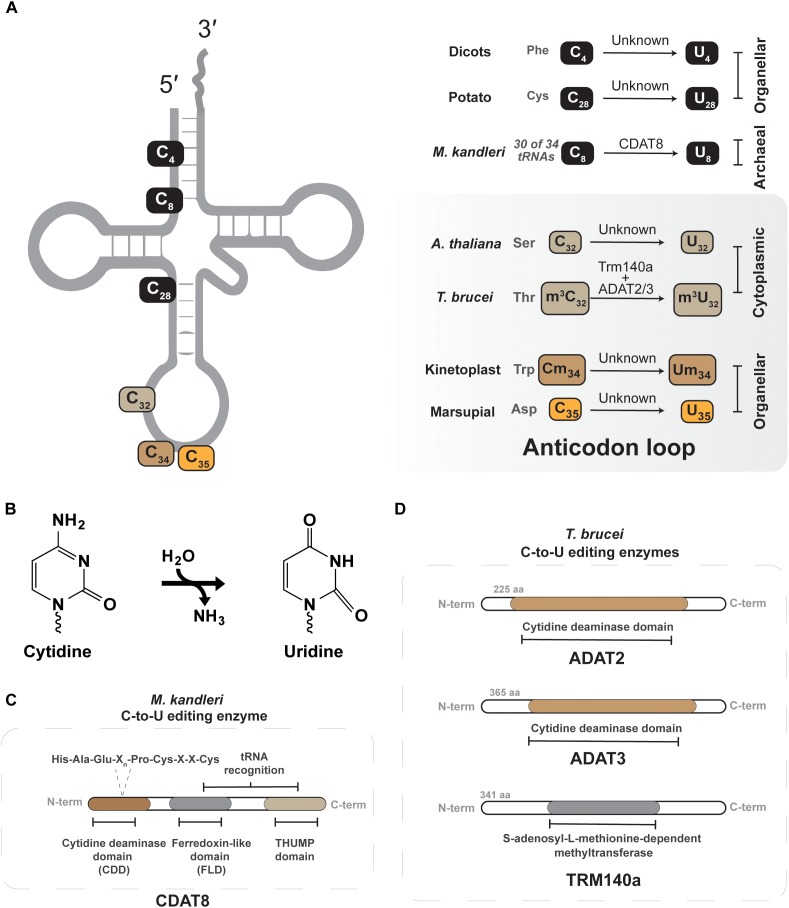
Cytidine-to-uridine (C-to-U) editing of tRNAs. **(A)** Shows the different C-to-U edited nucleotide positions that have been described in different tRNAs of different organisms. The organism and the nucleotide position along with tRNA identity are shown in the right panel. The enzyme identity is presented over the arrow. The gray panel denotes all known C-to-U editing events occurring in the anticodon loop. **(B)** Shows the C-to-U deamination reaction. **(C)** Depicts the conserved active-site residues in the tRNA C-to-U deaminase from archaea (CDAT8). **(D)** Depicts the active-site domain of both the ADAT2/3 and Trm140a of *T. brucei*, these enzymes interdependently.

Certain archaea and eukaryotes contain C-to-U edited tRNAs, but in many instances the enzymes responsible remain to be identified (Figure 3) (Janke and Paabo, 1993; Marchfelder et al., 1996; Alfonzo et al., 1999; Fey et al., 2002; Randau et al., 2009; Grewe et al., 2011). In the archaeon *Methanocryptus kandleri*, CDAT8, a cytidine deaminase homolog, catalyzes C-to-U conversion at position 8 of tRNAs (Randau et al., 2009). For many tRNAs an encoded U at position 8 forms a Hoogsteen base pair with A_14_ to assist in the proper folding of mature L-shaped tRNAs (Romby et al., 1985). However, 30 out of 34 tRNAs that contain A_14_ in *M. kandleri*, are encoded with a cytidine at position 8 that must be deaminated by CDAT8 to form U_8_ (Randau et al., 2009) and ensure proper tRNA folding. The reason for having such a large number of tRNAs that require C-to-U editing in *M. kandleri*, rather than encoding for U_8_-containing transcripts remains obscure. One probable answer might be the extreme environment in which *M. kandleri* lives, which favors G:C pairing in the DNA for optimal genome stability, yet still requires C-to-U editing for proper tRNA folding (Randau et al., 2009).

C-to-U editing of tRNAs in eukarya was first reported in protists, plants and marsupial mitochondria (Janke and Paabo, 1993; Lonergan and Gray, 1993a,b; Marechal-Drouard et al., 1996). However, the marsupial system provided the first example of C to U editing at the anticodon nucleotides. These organisms do not encode a tRNA for decoding mitochondrial aspartate codons. To solve this issue, the anticodon of tRNA^Gly^_G_**_C_**_C_ is C-to-U edited to tRNA^Asp^_G_**_U_**_C_, which is recognized by the mitochondrial aspartyl tRNA synthetase to produce a functional ortholog to tRNA^Asp^ (Janke and Paabo, 1993; Borner et al., 1996). This quirk of marsupial mitochondria paves the way for identification of similar mechanisms in nature, where an encoded tRNA gene can be edited to function as an alternative aminoacyl acceptor.

*Leishmania tarentolae* and *Trypanosoma brucei*, representative kinetoplastids, offer the only other example of anticodon C-to-U editing in tRNA (Alfonzo et al., 1999; Charriere et al., 2006). As in other organisms, the mitochondrial genome lacks certain tRNA genes that must be actively imported from the cytoplasm (Paris et al., 2009). In some instances, the mitochondrial translational code is not compatible with nuclear encoded tRNAs, as is the case for decoding UGA as tryptophan in mitochondria, not a stop codon (Barrell et al., 1979). In *L. tarentolae* and *T. brucei* UGA is used as a tryptophan codon in mitochondria, while the nucleus only contains a single-copy tRNA^Trp^**_C_**_CA_ to decode the canonical UGG codons. After import of tRNA^Trp^**_C_**_CA_ into the mitochondria, position 34 is C-to-U edited to create tRNA^Trp^**_U_**_CA_ as part of the mechanism that reassigned the UGA codons from stop to tryptophan. The enzyme responsible for this essential editing event is still unknown, but one would guess a deamination mechanism (Alfonzo et al., 1999; Charriere et al., 2006). Interestingly, only approximately 40–50% of the tRNA^Trp^ is edited after transport to the trypanosome mitochondria, raising questions as to how this balance is kept. The answer partly rests on the unusual thiolation at U_33_ in this tRNA, which negatively impacts C-to-U editing of C_34_ (Wohlgamuth-Benedum et al., 2009).

In the mitochondria of dicotyledon plants, C-to-U editing takes place outside the anticodon region and does not directly influence the decoding capacity of tRNAs (Marechal-Drouard et al., 1996; Fey et al., 2002; Ichinose and Sugita, 2016). In potato mitochondria, C-to-U editing corrects a mismatch-encoded pair C_4_:A_69_ in 5′ processed pre-tRNA^Phe^_GAA_ to U_4_:A_69_ (Binder et al., 1994; Marechal-Drouard et al., 1996). After editing 5′ processed pre-tRNA^Phe^_GAA_ can properly fold resulting in efficient removal of the 3′ trailer by mitochondrial RNaseZ (Kunzmann et al., 1998). In a modeling study of quillwort, *Isoetes engelmannii*, extensive C-to-U editing was predicted at 43 possible tRNA sites (Grewe et al., 2011; Ichinose and Sugita, 2016). The author′s obtained cDNA sequence data for 36 such sites, and among them 29 showed C-to-U editing (Grewe et al., 2011). Interestingly four sites showed U-to-C conversion, invoking the necessity of a likely transamination reaction (Grewe et al., 2011). There are other thematically similar C-to-U editing events that occur for tRNAs in plant mitochondria, and these are well-reviewed elsewhere (Fey et al., 2002; Paris et al., 2012).

Position 32 of the anti-codon stem loop is another site where tRNAs from multiple domains contain C-to-U conversions. It was first described in *T. brucei* that all three tRNA^Thr^ isoacceptors undergo C-to-U editing at position 32 (Rubio et al., 2006; Gaston et al., 2007). *Trypanosoma brucei* ADAT2/ADAT3, previously discussed in the context of A-to-I editing at position 34, has been shown to perform C-to-U editing of tRNA^Thr^_AGU_ at position 32 (Rubio et al., 2007; Rubio et al., 2017). However, this editing event first requires methylation at this site, discussed in more detail in Section “m^3^C-to-m^3^U.” Within the ADAT2/ADAT3 heterodimeric complex, the C-terminal region of ADAT2 assists in tRNA binding, while ADAT3 provides a structural role in formation of the catalytic deaminase core (Rubio et al., 2007), similar in arrangement as the two subunit methyltransferases discussed in Section “Interdependent Methyltransferases.” The biological significance of this editing is not exactly clear, but some evidence showed it is important for protein synthesis (Rubio et al., 2017), but C_32_ methylation and editing do not impact aminoacylation efficiency. Given its position in the anticodon loop, likely such effects in protein synthesis may be due to some function in translational efficiency or accuracy. Similar editing events have been recently described in *Arabidopsis thaliana*, where tRNA^Ser^_AGA_ and tRNA^Ser^_GCU_ are C-to-U edited at position 32 in the nucleocytoplasmic compartment by a presently unknown enzymatic mechanism (Zhou et al., 2014).

## Interdependent Methylation and Editing and Its Biological Relevance

### m^1^I Formation in Eukaryotes Versus Archaea

As discussed in Section “Adenosine to Inosine (A-to-I) Editing” A-to-I editing is found at three tRNA sites: position 34, 37, and 57. Intriguingly, inosines at position 37 and 57 can be methylated to form m^1^I_37_ and m^1^I_57_, by distinct chemical pathways (Yamaizumi et al., 1982; Grosjean et al., 1995, 1996). At position 37, after ADAT1 converts A-to-I, SAM-dependent Trm5 can act directly on N-1 of inosine (Gerber et al., 1998; Maas et al., 1999; Brule et al., 2004; Macbeth et al., 2005). As Trm5 is essential in most organisms catalyzing the generation of m^1^G_37_, the biological significance of its involvement in the modification of edited I_37_ remains unclear (Paris et al., 2013). The opposite order of events occurs at position 57 in archaea, where a SAM-dependent TrmI-family member must first methylate A_57_ before it becomes a substrate for deamination to inosine (Yamaizumi et al., 1982; Grosjean et al., 1995, 1996). The enzyme, or enzymatic complex, responsible for m^1^A_57_-to-m^1^I_57_ has not been identified, and the biological significance of this modification remains to be articulated. However, methylation followed editing also occurs in specific m^3^C-to-m^3^U conversions.

### m^3^C-to-m^3^U

**FIGURE 4:**
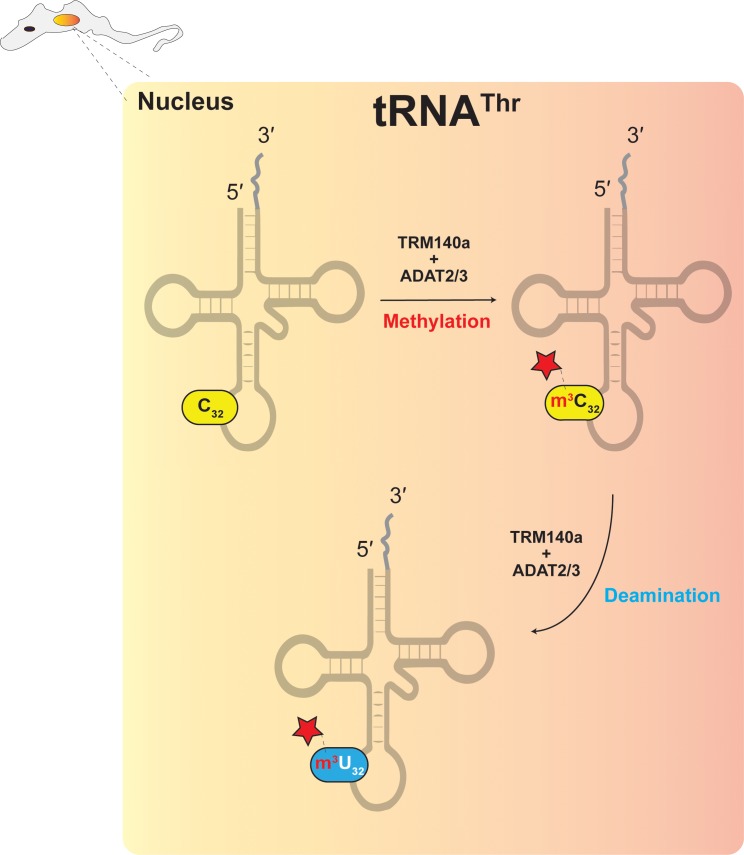
The interdependence model showing the necessary connection between RNA modification and RNA editing.

In trypanosomes, down regulation of ADAT2/ADAT3 expression reduces A_34_-to-I_34_ editing in tRNA^Thr^_AGU_ at position 34 and C-to-U editing at position 32 (Rubio et al., 2007). It was originally hypothesized that ADAT2/ADAT3, which has clear sequence similarity with cytidine deaminases, may be responsible, however, initial attempts to reconstitute C-to-U editing with *T. brucei* ADAT2/ADAT3 were not successful (Rubio et al., 2007). Since C_32_ of tRNA^Thr^_AGU_ is methylated to form m^3^C_32_, it suggested that C-to-U editing could require methylation prior to deamination (Figure 4) (Rubio et al., 2017), as discussed previously for archaeal m^1^A_57_-to-m^1^I_57_. The methyltransferase Trm140 was later identified as responsible for formation of m^3^C_32_ (D’Silva et al., 2011; Noma et al., 2011) and true to expectations, Trm140 and ADAT2/ADAT3 were shown to work interdependently to convert m^3^C_32_ to m^3^U_32_ *in vitro* (Rubio et al., 2017). Further experimentation also showed that Trm140 and ADAT2/ADAT3 are likely to work sequentially and as a complex (Rubio et al., 2017). How these two enzymes converge simultaneously on a single RNA substrate and act at the same nucleotide position remains an open question. However, recent studies demonstrated that the two enzymes bind their tRNA substrate synergistically, whereby binding affinity increases significantly if both proteins are present in the reaction (McKenney et al., 2018).

ADAT2/ADAT3 can deaminate DNA *in vitro* and *in vivo*, this mutagenic activity is dampened through association with Trm140 (Rubio et al., 2007, 2017). By extension a similar mechanism, may inform how the cytidine deaminase, AID, specifically targets genes that encode immunoglobulin receptors while leaving the rest of the genome unaffected during B cell somatic hypermutation (Teng and Papavasiliou, 2007). It is likely that additional protein factors influence specificity of AID toward its substrate genetic loci.

## Concluding Remarks

Much has been written about the mechanisms that lead to, and determine the fate of, duplicated genes. It is clear that once gene duplication occurs, one copy is free to mutate via genetic drift, to accumulate mutations perhaps by a “neutral evolutionary ratchet” (Covello and Gray, 1993; Gray et al., 2010; Lukes et al., 2011). These mutations, in turn, can lead to total loss of function of the duplicated gene and the creation of pseudogenes. Alternatively, and more importantly, duplication may provide a powerful route to neofunctionalization or subfunctionalization of genes (Stoltzfus, 1999). The former makes the mutated duplicate gene acquire new functions different from that of the ancestral gene; the latter leads to a partitioning of labor so that each copy now carries a subset of the functions originally performed by the ancestral state. In this review, we have focused on various examples of modification enzymes, where evolution has pushed the system into one of the categories above. For example, in the case of the Trm61/Trm6 methyltransferase, sequence comparisons strongly suggest that these are paralogs. Here, the neutral acquisition of mutations without obvious gains in fitness led to a level of divergence that at some point may have become important in expanding substrate specificity, thus diversifying the number of targets the new enzyme could methylate.

In the case of Trm7/Trm732 and Trm7/Trm734, no evidence exists for gene duplication; neither Trm732 nor Trm734 have significant sequence conservation with known methyltransferases. Instead, they share similarity with other protein families, for example Trm734 with WD-domain family proteins. It may be that independent stochastic mutations accumulated in each gene for evolution of a dimerization interface that allowed each protein to interact with the Trm7 partner. In doing so, differential complex formation permitted a novel division of labor; one complex now targets position 32 of tRNAs and the other position 34. This is, of course, assuming that the catalytic subunit Trm7 derives from an ancestral gene that at some point was able to efficiently methylate both positions.

Similar arguments can be made with the tRNA A-to-I deaminase of trypanosomes, TbADAT2/TbADAT3, a heterodimeric enzyme comprised of two subunits encoded by clear paralogs (Rubio et al., 2007). Accumulation of mutations in one or both copies may have forced each paralog to strictly rely on the other for activity. Such cases echo themes observed in enzyme-prozyme complexes (Nguyen et al., 2013; Volkov et al., 2016), where in polyamine biosynthesis a catalytically dead paralog regulates the activity of an active paralogous enzyme. We (Gaston et al., 2007; Rubio et al., 2007), and others (Elias and Huang, 2005) have argued, that at least in the case of ADAT2/ADAT3 in eukaryotes, gene duplication led to the expansion in the specificity of the enzyme toward more molecular substrates. For example, ADATa, the homologous enzyme from bacteria is active as a homodimer, targets a single tRNA^Arg^ *in vivo* and a “minimalist” tRNA^Arg^ anti-codon stem loop is a sufficient substrate *in vitro* (Wolf et al., 2002). Not surprisingly the co-crystal structure of ADATa shows that residues near or at the active site are necessary for RNA binding (Losey et al., 2006). The eukaryotic counterpart ADAT2/ADAT3 recognizes seven to eight different tRNAs depending on the organism, but is only active on full length tRNAs (Auxilien et al., 1996). Years ago, we showed that one of the critical RNA binding domains of the *T. brucei* enzyme lies at the C-terminus of one subunit and that residues near the active site minimally contribute to substrate binding (Ragone et al., 2011). Thus, movement of critical binding residues away from the active site increased active site flexibility to allow for the observed expansion in substrate specificity. Again, such subtle, yet important, evolutionary changes in eukaryotic tRNA deaminases are only made possible by gene duplication and the previously proposed effects of “constructive neutral evolution” (Covello and Gray, 1993; Stoltzfus, 1999).

Finally let’s consider m^3^C/m^3^U editing and methylation at position 32 of several tRNAs in *T. brucei* (Figure 4) (also described in plants). The TbTrm140 m^3^C methyltransferase does not result from an obvious duplication of a deaminase gene and *vice versa*. While both enzymes come together to form a stable, active complex in the nucleus, the TbADAT2/TbADAT3 heterodimer is also active in the cytoplasm as a free enzyme catalyzing essential A-to-I deaminations (Rubio et al., 2017). In this particular case, both enzymes accumulated mutations in a neutral fashion, likely independent of each other. The question is how could TbTrm140 accumulate mutations without causing deleterious effects on the organism. The answer may involve TbMtase37, a paralog of Trm140 within the *T. brucei* genome, of currently unknown function (Fleming et al., 2016). This paralog might provide the necessary duplicate and essential function, which allowed TbTrm140 to mutationally drift and neofunctionalize with a seemingly unrelated enzyme like TbADAT2/TbADAT3 to modify and edit new substrates. What makes this case unusual is the fact that both enzymes have all the conserved residues required for activity and both may be active on other substrates, yet by themselves are totally inactive for methylation and deamination of tRNA^Thr^ position 32 of *T. brucei*. We thus introduce the concept of “enzyme co-activation,” whereby enzymes active with some substrates but inactive with others, gain new function upon their association and indeed co-activate each other.

Neo- or sub-functionalization includes a combination of non-adaptive and, later, adaptive mutations as originally suggested by Gray et al. (2010) Regardless of what factors or mechanisms are at play, the question then remains: Are there fitness gains to be made by organisms by the examples in this review? At least in trypanosomes, we have long appreciated the interdependent nature of RNA editing and modification, under the hypothesis that these events fine-tune translation to the ever-changing environmental conditions during the life cycle of these parasites (Paris et al., 2012). Interdependent modification and editing may serve to maintain levels of edited and unedited tRNAs in response to changes in environment or life stages. The same could be true of many other modifications and subsequent use of alternative substrates (Helm and Alfonzo, 2014); enzyme co-activation may provide an additional level of “tunability” to ensure fast responses to ever changing growth conditions, not only in response to stress, but also in maintenance of general cell homeostasis.

## Author Contributions

All authors listed have made a substantial, direct and intellectual contribution to the work, and approved it for publication.

## Conflict of Interest Statement

The authors declare that the research was conducted in the absence of any commercial or financial relationships that could be construed as a potential conflict of interest.
